# Carelessness

**Published:** 1854-06

**Authors:** 


### CARELESSNESS.

If you want to travel through the world in a quiet, contented way, don't get careless in any respect. Man, in every phase of life, is particularly given to carelessness. If he is on the high road to wealth and station, he becomes careless of those who perhaps were the very means of his good fortune. On the other hand, if he is unfortunate in business he loses his self-respect, and rushes to the dram shop or gaming table.

Whatever position a man finds himself placed in, whether by accident, fortunate speculation, or persevering industry, he should always retain that command over himself that will entitle him to the good will of old as well as new friends. If a man rises from comparative obscurity to some degree of eminence of any kind, and with no intention to offend, but carelessly notices an old friend if he meet him, he is very likely to get the ill-will of his more humble but old associate. The first time this careless recognition is noticed it produces a bad effect, and the next dislike, and finally hatred and contempt. We have known some of the very best friends in the world completely estranged by a wrong interpretation of acts towards each other.

It is a common belief that as a man advances in the world he is desirous of *cutting* those who do not gain so rapidly as himself. This is an error, no doubt, in many instances, and the remedy is one of the easiest things in the world. A little of the starch out of the one, and the slightest liberal feeling on the other, will be found to be a true panacea for nine-tenths of the imaginary shys which lead to the entire separation of old friends, and even goes so far sometimes as to produce bad feelings among relatives.

We shall end this brief article on carelessness by repeating the advice with which we begun. If you want to travel through the world in a quiet contented way, don't get careless in any respect. Be free with your friends as though no change affected your condition in life, let that condition have changed ever so much, be it for better or worse.—*Yonkers Herald.*

